# Defining the psychiatric and financial burden of mental and substance use disorders in cancer patients

**DOI:** 10.1002/cam4.5548

**Published:** 2022-12-19

**Authors:** Sujith Baliga, Brett Klamer, Joshua D. Palmer, Sharla Wells‐Di Gregirio, Sachin S. Kale, Marcelo Bonomi, Matthew O. Old, James W. Rocco, Dukagjin M. Blakaj

**Affiliations:** ^1^ Department of Radiation Oncology The Ohio State University Wexner Medical Center Columbus Ohio USA; ^2^ Department of Biomedical Informatics, Center for Biostatistics The Ohio State University Columbus Ohio USA; ^3^ Department of Internal Medicine, Division of Palliative Medicine The Ohio State University Wexner Medical Center Columbus Ohio USA; ^4^ Department of Medical Oncology The Ohio State University Wexner Medical Center Columbus Ohio USA; ^5^ Department of Otolaryngology‐Head and Neck Surgery The Ohio State University Wexner Medical Center Columbus Ohio USA

**Keywords:** cancer, mental health, substance use

## Abstract

**Purpose:**

To identify the proportion of Emergency Department (ED) visits in cancer patients associated with a mental and substance use disorder (MSUD) and the subsequent healthcare costs.

**Methods:**

Nationally representative data on ED visits from 2009 to 2018 was obtained from the Nationwide Emergency Department Sample (NEDS). We identified cancer‐related visits with or without a MSUD using the Clinical Classifications Software diagnoses documented during the ED visit. Survey‐adjusted frequencies and proportions of ED visits among adult cancer patients with or without a MSUD was evaluated. Survey‐adjusted multivariable logistic regression models were used to examine demographic and clinical predictors of the presence of an MSUD and the likelihood of hospital admission for patients with a primary MSUD.

**Results:**

Among 54,004,462 ED visits with a cancer diagnosis between 2009 and 2018, 11,803,966 (22%) were associated with a MSUD. Compared to a primary diagnosis of cancer, patients who presented to the ED with a chief complaint of MSUD were more likely to be female (54% vs. 49%), younger (median: 58 vs. 66), more likely to have Medicaid insurance, and more likely to be discharged home. The three most common MSUD diagnoses among cancer patients were alcohol‐related disorders, anxiety disorders, and depressive disorders. The total costs associated with a primary MSUD from 2009 to 2018 was $3,133,432,103, of which alcohol‐related disorders claimed the largest majority. Younger age (OR per 10‐year increase: 0.86, 95% CI: 0.85, 0.86) and female sex (OR: 1.34, 95% CI: 1.33–1.35) were associated with higher odds of having an MSUD.

**Conclusions:**

Our findings demonstrate a high burden of psychiatric and substance use illness in the cancer population and provide the rationale for early psychosocial intervention to support these patients.

## Abstract 2

These findings demonstrate a high rate of mental and substance use disorder (MSUD) in cancer patients with one in five cancer‐related visits to the ED presenting with a MSUD. Younger age and female gender were more likely associated with mental or substance use disorders. Healthcare costs for MSUD were 3 billion dollars over 10 years.

## 1 INTRODUCTION

The prevalence of cancer survivors in the United States is expected to significantly grow in the next decade, from 16.9 million Americans in 2019 to 22 million by 2030.^1^ In addition, cancer survival has continued to improve over the last three decades, due to significant technological advances which have improved the delivery of chemotherapy, radiotherapy, and surgery. Nevertheless, the sequalae and side effects of multimodality treatment are often lifelong and impact both the psychological and physiological health of cancer survivors. Cancer treatment can have a profound psychological impact on these patients, which can significantly impact their mental health, as well as their overall quality of life. A recent meta‐analysis demonstrated that the prevalence of mood disorders such as depression and anxiety among long‐term cancer survivors was between 10%–12%.^2^ Although treatment of mental health disorders is increasingly being recognized as an important part of cancer survivorship, there is an incomplete understanding of the landscape of mental health disorders in the cancer population. While many studies have reported on the prevalence of mental illness in cancer patients, few studies have evaluated trends in emergency room visits, hospitalizations, and the overall cost associated with mental health in the cancer population. A detailed analysis of mental health hospitalizations and cost in cancer patients could provide an opportunity to develop strategies to address this vital aspect of survivorship. The objectives of this study were to use the Nationwide Emergency Department Sample (NEDS) database to evaluate the frequency of mental health disorders among adult patients with cancer in the ED setting and to examine the characteristics related to inpatient admission in this population.

## 2 METHODS

### 2.1 Study design and sample

The Nationwide Emergency Department Sample (NEDS) was developed for the Healthcare Cost and Utilization Project (HCUP) and produces national estimates of hospital owned ED visits. The NEDS is the largest all‐payer ED database in the United States and contains information from over 40 million ED visits. The database was exempted from IRB approval as the data are classified as a limited dataset and covered under a data use agreement to ensure privacy. This study evaluated national estimates of Emergency Department (ED) visits among adult cancer patients with a mental and substance use disorders from 2009 to 2018. The unit of analysis provided by NEDS is the emergency department discharge record, not a person or patient.

We identified all adult (≥18) patients with cancer‐related visits using the Clinical Classifications Software (CCS) diagnosis codes reported for each ED visit. The CCS is based on the International Classification of Disease, 9th Revision, Clinical Modification (ICD‐9 CM) and recodes the 14,000 diagnosis codes into a smaller number of 260 meaningful disease categories. To identify patients with a specific diagnosis of cancer, CCS codes 11–45 were included. These codes do not necessarily represent patients who are receiving active treatment or those with recent diagnoses. The codes reflect the ICD‐9 diagnosis codes that were placed by the medical team at the time of the ED visit. For ED visits, the first listed diagnosis represents the chief reason for the ED services provided.^3^ The secondary diagnoses are conditions that coexist at the time of the ED visit or inpatient admission and affect the treatment or management of the care received by the patient. The CCS code categorized as “Secondary Malignancies” includes patients with secondary malignancies of the lymph nodes, lung, liver, brain/spine, and bone. Once cancer patients were identified, the CCS was used to identify mental health and substance abuse cases using a software that converts the ICD9 and ICD10 codes to CCS codes. The categories created were alcohol‐related disorders, anxiety disorders, bipolar disorders, cannabis‐related disorders, depressive disorders, disruptive, impulse control, and conduct disorders, miscellaneous mental disorders, miscellaneous substances and addictive disorders, obsessive–compulsive disorders, opioid‐related disorders, personality disorders, schizophrenia and related disorders, sedative‐related disorders, somatic symptom disorders, suicidal ideation or attempt, and trauma and stressor‐related disorders. Additional variables that were analyzed include age, sex, vital status, income quartile, insurance status, age, total charges, hospital region, and disposition (discharged vs. admitted).

### 2.2 Statistical analysis

NEDS file specifications were used to prepare and read in comma‐separated core, hospital weights, and emergency department files for the years 2009 through 2018 using R version 4.1.0 and the “data. Table” (version 1.14.0) package. The “icd” (version 4.0.9) package was used to convert ICD‐10 diagnosis codes to CCS codes for years 2015q4 through 2018. ICD‐9 and ICD‐10 codes for mental/substance use disorder were obtained from HCUP statistical briefs.^4^, ^5^ The “touch” (version 0.1–5) package was used to crosswalk these ICD‐9 and ICD‐10 codes and resulted in our mental/substance use disorder definitions. Each record in NEDS includes up to 35 unique diagnosis codes, and each of those codes was evaluated for CCS and MSUD classification, the first of these being summarized in our results.

NEDS is a stratified, single‐stage cluster sample design. All analyses were based on weighted data that adjusted for the NEDS' survey design to produce discharge‐level estimates using R packages “survey” (version 4.1–1) and “srvyr” (version 1.1.0). Descriptive statistical analyses consisted of survey‐adjusted frequencies, percentages, medians, and 95% confidence intervals (CI). A survey‐adjusted logistic regression models was fit on all records for the outcome of any indicated MSUD diagnosis versus not and a second model was fit only for records with primary MSUD for the outcome of admission as inpatient versus not. Each logistic model included predictor variables for age, sex, income quartile, primary payer, and cancer type. Age was scaled by 10 to produce coefficient estimates corresponding with a per 10‐year change and both models assumed additive, linear relationships on the log‐odds scale. Complete case analysis was used for all analyses.

## 3 RESULTS

### 3.1 Characteristics of study cohort

There were 54,004,462 ED visits associated with a cancer diagnosis between 2009 and 2018, of which 11,803,966 (22%) were associated with a mental and substance use disorder (MSUD). Table 1 shows a comparison of demographic, socioeconomic, and discharge characteristics of cancer patients with and without a MSUD. Cancer patients with an associated MSUD diagnosis during the ED visit were likely to be younger (median age 67 vs. 71), female (60% vs. 52%), have Medicaid insurance (14% vs. 9.3%), and more likely to be admitted as an inpatient (68% vs. 51%). Table 2 shows a comparison of ED visits for cancer (*n* = 5,302,556) or MSUD (*n* = 1,002,923) as the chief presentation. Compared to visits primarily for cancer, those admitted to the ED for a MSUD were more likely to be female (54% vs. 49%), younger (median age 58 vs. 66), less likely to die in the hospital (0.6% vs. 6.1%), more likely to have Medicaid insurance (22% vs. 15%), and more likely to be discharged home (40% vs. 17%).

**TABLE 1:** Clinical and demographic characteristics of ED visits from adult cancer patients who presented to the ED with or without an associated MSUD diagnosis from 2009 to 2018

Characteristics	No MSUD, *N* = 42,200,496^a^	MSUD, *N* = 11,803,966^a^
Age at ED visit (Years)	71 (59, 81)	67 (55, 78)
Sex		
Female	21,763,627 (52%)	7,071,041 (60%)
Male	20,429,595 (48%)	4,730,631 (40%)
(*N* missing)	7273	2294
Vital status		
Alive	40,698,569 (97%)	11,442,153 (97%)
Died in hospital	1,135,560 (2.7%)	296,140 (2.5%)
Died in ED	137,054 (0.3%)	11,241 (<0.1%)
(*N* missing)	229,313	54,431
Total charge for visit (Dollars)	2265 (1307, 4239)	2159 (1307, 3777)
(*N* missing)	8,658,345	2,644,028
Primary payor		
Medicare	27,232,911 (65%)	7,368,946 (62%)
Private insurance	8,718,109 (21%)	2,109,323 (18%)
Medicaid	3,904,174 (9.3%)	1,634,827 (14%)
Self‐pay	1,288,774 (3.1%)	381,832 (3.2%)
Other	911,196 (2.2%)	265,542 (2.3%)
No charge	101,781 (0.2%)	32,194 (0.3%)
(*N* missing)	43,552	11,303
Disposition of patient		
Admitted as inpatient	21,665,253 (51%)	7,995,798 (68%)
Routine	18,198,124 (43%)	3,265,643 (28%)
Other	2,337,119 (5.5%)	542,525 (4.6%)
Hospital region		
South	15,786,039 (37%)	4,451,678 (38%)
Midwest	9,825,067 (23%)	2,962,111 (25%)
West	8,744,900 (21%)	2,194,372 (19%)
Northeast	7,844,490 (19%)	2,195,804 (19%)
Income quartile		
Quartile 1 (0–25th percentile)	11,064,607 (27%)	3,263,037 (28%)
Quartile 2 (26–50th percentile)	10,623,993 (26%)	3,013,238 (26%)
Quartile 3 (51–75th percentile)	9,988,962 (24%)	2,736,028 (24%)
Quartile 4 (76–100th percentile)	9,724,318 (23%)	2,555,671 (22%)
(*N* missing)	798,615	235,993
Most frequent cancer (Top 5)		
Breast cancer	6,025,168 (14%)	1,866,900 (16%)
Prostate cancer	4,724,027 (11%)	896,810 (7.6%)
Lung cancer	3,746,049 (8.9%)	1,108,959 (9.4%)
Secondary malignancies	3,414,332 (8.1%)	992,991 (8.4%)
Colon cancer	3,134,840 (7.4%)	761,553 (6.5%)

^a^ *n* (%); Median (IQR).

**TABLE 2:** Comparison of clinical and demographic characteristics for ED visits associated with a primary diagnosis of cancer or MSUD from 2009 to 2018

Characteristics	Cancer, *N* = 5,302,556^a^	MSUD, *N* = 1,002,923^a^
Age at ED visit (Years)	66 (56, 77)	58 (48, 69)
Sex		
Male	2,682,755 (51%)	458,792 (46%)
Female	2,618,344 (49%)	543,962 (54%)
(*N* missing)	1457	170
Vital status		
Alive	4,946,687 (94%)	990,613 (99%)
Died in hospital	323,466 (6.1%)	6234 (0.6%)
Died in ED	14,653 (0.3%)	253 (<0.1%)
(*N* missing)	17,751	5824
Total charge for visit (Dollars)	1972 (1228, 3331)	2005 (1155, 3557)
(*N* missing)	1,208,151	208,677
Primary payor		
Medicare	2,888,666 (55%)	481,667 (48%)
Private insurance	1,244,861 (24%)	200,300 (20%)
Medicaid	770,532 (15%)	216,955 (22%)
Self‐pay	232,789 (4.4%)	68,664 (6.9%)
Other	135,404 (2.6%)	28,265 (2.8%)
No charge	24,457 (0.5%)	5635 (0.6%)
(*N* missing)	5847	1437
Disposition of patient		
Admitted as inpatient	4,157,599 (78%)	507,862 (51%)
Routine	918,512 (17%)	397,883 (40%)
Other	226,445 (4.3%)	97,179 (9.7%)
Hospital region		
South	2,106,912 (40%)	357,256 (36%)
Midwest	1,095,541 (21%)	237,094 (24%)
West	1,001,452 (19%)	199,440 (20%)
Northeast	1,098,651 (21%)	209,134 (21%)
Income quartile		
Quartile 1 (0–25th percentile)	1,503,380 (29%)	287,655 (30%)
Quartile 2 (26–50th percentile)	1,328,146 (26%)	252,237 (26%)
Quartile 3 (51–75th percentile)	1,223,565 (24%)	227,295 (23%)
Quartile 4 (76–100th percentile)	1,129,415 (22%)	205,342 (21%)
(*N* missing)	118,050	30,394

^a^ *n* (%); Median (IQR).

There were 6487 visits that were associated with a primary MSUD and which resulted in death either in the hospital (96.1%) or ED (3.9%). Of the 6487 visits, 4220 (65%) were associated with male sex and the median age was 62 years. The majority of visits were from the two lowest income quartiles (57%) and only 18% were from the highest income quartile. The three most common cancer types associated with a primary MSUD and death were cancer of the liver and intrahepatic bile duct (21%), secondary malignancies (16%), and cancer of the bronchus; lung (9.0%). The three most common primary MSUD in this patient group were alcohol‐related disorders (51%), suicidal ideation or attempt (22%), and opioid‐related disorders (15%).

### 3.2 Frequency of primary mental and substance use disorder ED visits and association with cancer

**FIGURE 1:**
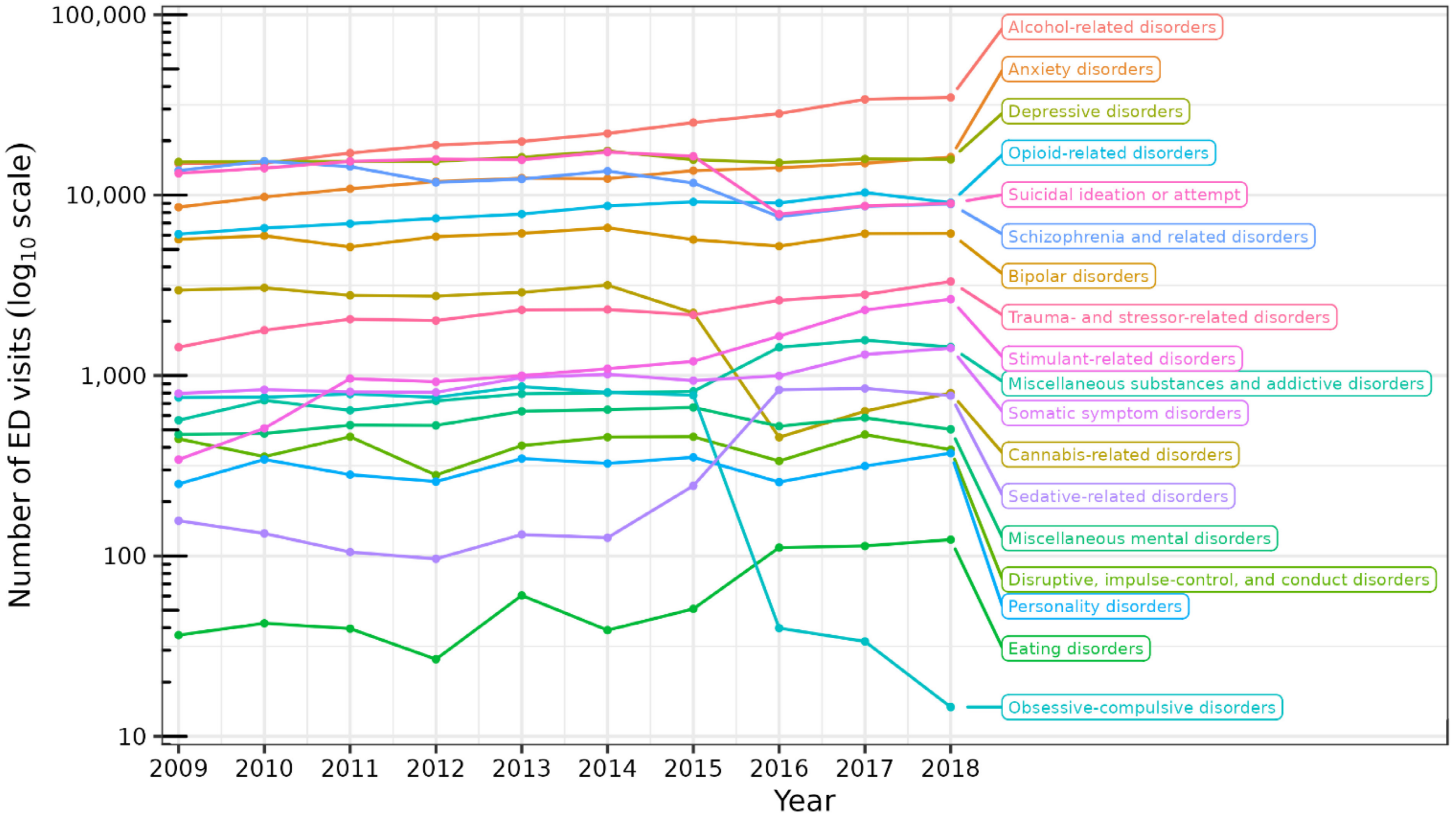
Number of ED visits associated with a primary MSUD from 2009 to 2018.

**FIGURE 2:**
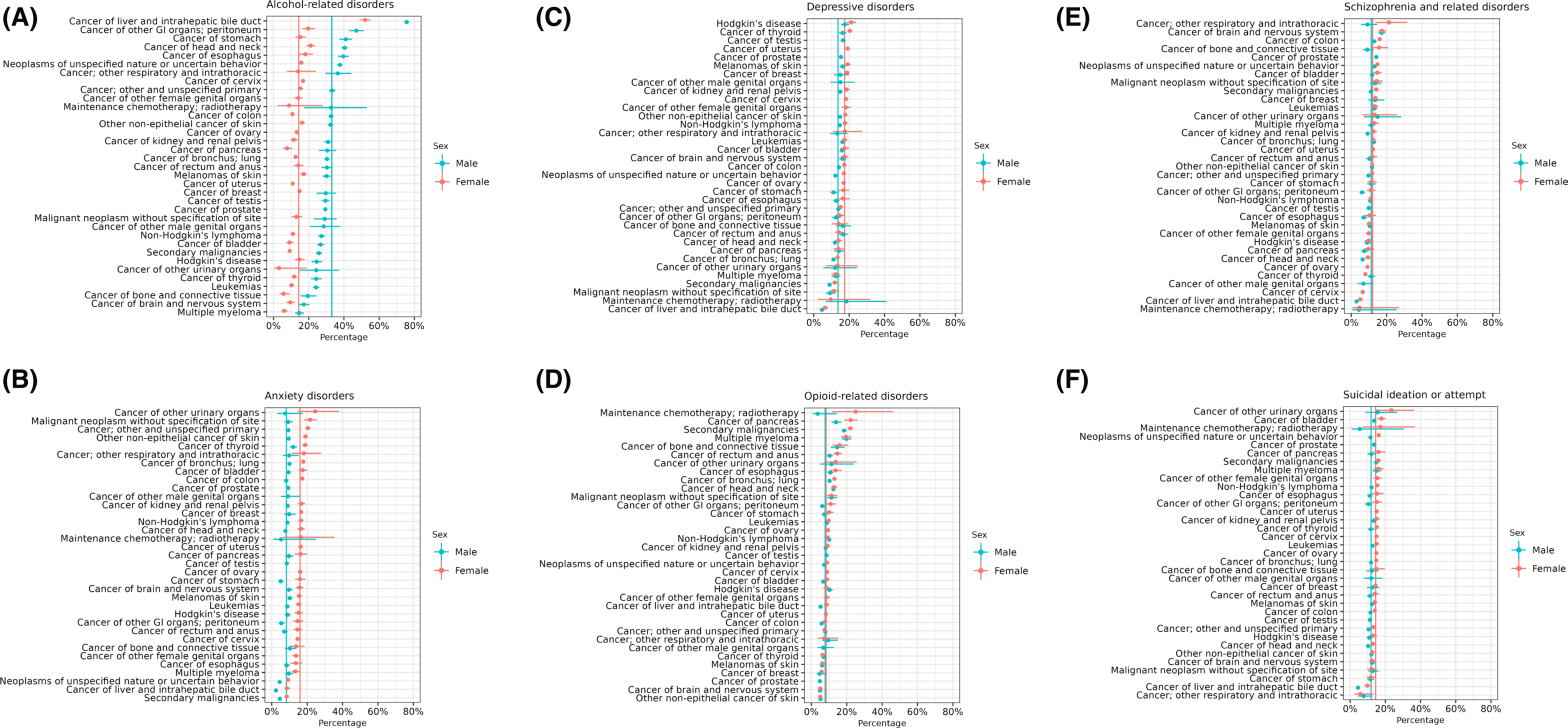
The percentage of ED visits with primary MSUD (A) alcohol‐related disorders, (B) anxiety disorders, (C) depressive disorders, (D) opioid‐related disorders, (E) schizophrenia and related disorders, and (F) suicidal ideation or attempt within each cancer type. Horizontal lines through each point represent the 95% CI. Vertical lines represent the overall percentage of ED visits across all cancer types for each of the top six primary MSUD categories when stratified by sex (female in red and male in cyan). Points to the right of each sex's vertical line may indicate higher than expected likelihood of MSUD for the associated cancer type, and points to the left of each sex's vertical line may indicate lower than expected likelihood of MSUD for the associated cancer type. Cancer type was ordered based on the sex whose overall MSUD percentage was greatest.

Figure 1 shows the yearly frequency of ED visits associated with different primary MSUD categories in patients who had cancer. The six most frequent primary MSUD diagnoses were alcohol‐related disorders, anxiety disorders, depressive disorders, opioid‐related disorders, schizophrenia and related disorders, and suicidal ideation or attempt. From 2009 to 2018, the frequency of alcohol‐related disorders increased 2.3 times and anxiety disorders increased 1.9 times, with a concomitant decrease in schizophrenia and related disorders. Figure 2 shows the percent of ED visits for these six primary MSUD categories within each cancer type and sex. Compared to other types of cancer, alcohol related disorders were most likely among visits with tumors of the liver, gastrointestinal tract, and head and neck. Compared to females, males' likelihood of primary alcohol‐related disorder was unexpectedly high among those with cancer of the stomach (Figure 2A). Anxiety disorders were most likely among females with cancer of other urinary organs and among all visits with thyroid tumors, non‐epithelial cancer of skin, and cancer of bronchus; lung (Figure 2B). Depressive disorders were more likely among visits with Hodgkin's disease, thyroid tumors, and sex‐specific cancers of the testis, prostate, and uterus (Figure 2C). Opioid‐related disorders were more likely in visits with pancreatic cancer, secondary malignancies, multiple myeloma, and cancer of the bone and connective tissue. The likelihood of opioid‐related disorders was unexpectedly high for females with corresponding maintenance chemotherapy and/or radiotherapy while males had a lower than expected likelihood among this cancer group (Figure 2D). Schizophrenia and related disorders were more likely in visits with a cancer of the brain and nervous system, colon, and prostate. Females had higher than expected likelihood of schizophrenia and related disorders among those with cancer of bone and connective tissue and other respiratory and intrathoracic cancers (Figure 2E). Suicidal ideation or attempt was most likely to be associated with cancer of the bladder, prostate, other urinary organs, multiple myeloma, and secondary malignancies.

### 3.3 Costs associated with primary mental and substance use disorders

**FIGURE 3:**
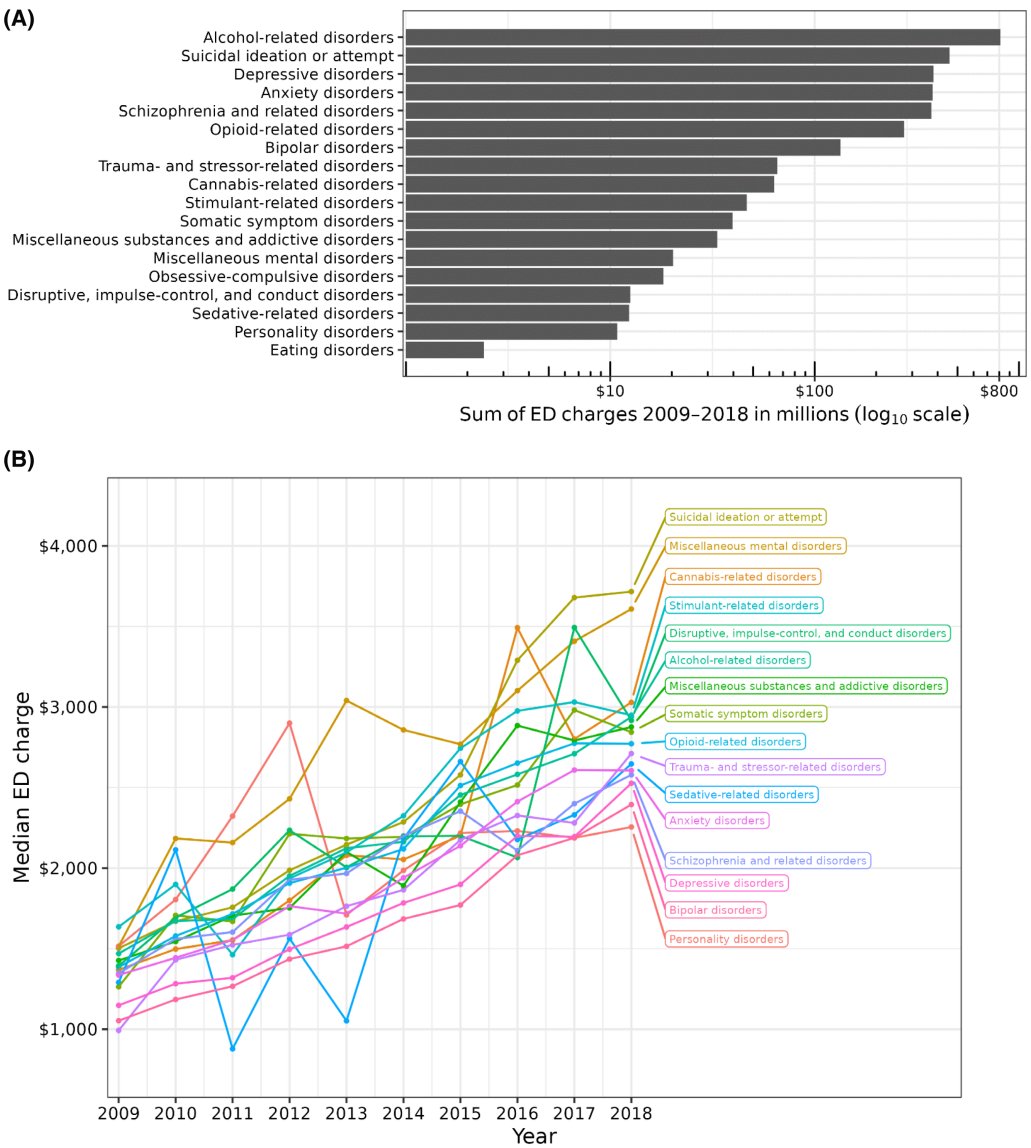
Cost associated with a primary MSUD by (A) total ED visit charges from 2009 to 2018 and (B) yearly median ED charge per visit. Due to low counts over time, eating disorders, and obsessive–compulsive disorders were excluded from Figure B.

The total costs associated with each primary MSUD category are shown in Figure 3. From 2009 to 2018, the total costs among all primary MSUD categories was $3,133,432, and alcohol‐related disorders, suicidal ideation or attempt, depressive disorders, and anxiety disorders made up 65% of all costs associated with an ED visit associated with a primary MSUD (Figure 3A). The costs associated with all primary MSUD categories increased from 2009 to 2018. (Figure 3B).

### 3.4 Demographic and clinical relationships with MSUD

The results of our multivariable logistic regression examining the relationship between demographic and clinical characteristics with presence of an MSUD is shown in Table 3. Younger age (OR per 10‐year increase: 0.86, 95% CI: 0.85, 0.86) and female sex (OR: 1.34, 95% CI: 1.33, 1.35) were associated with increased odds of MSUD. In overall comparison to the five other payor types, patients with private insurance (OR: 0.69, 95% CI: 0.68–0.70), self‐pay (OR: 0.83, 95% CI: 0.81–0.85), and other (OR: 0.97, 95% CI: 0.94–0.995) were less likely to have an MSUD. In overall comparison to other cancer types, the three cancers with greatest odds of any MSUD are cancer of liver and intrahepatic bile duct (OR: 1.52, 95% CI: 1.49, 1.56), other non‐epithelial cancer of skin (OR: 1.26, 95% CI: 1.23, 1.29), and head and neck cancers (OR: 1.20, 95% CI: 1.18, 1.22).

**TABLE 3:** Adjusted odds ratio estimates for presence of MSUD in ED visits from cancer patients during 2009–2018

Characteristics	OR	95% CI
Age at ED visit (per 10‐year increase)	0.86	**0.85, 0.86**
Sex		
Male	—	—
Female	1.34	**1.33, 1.35**
Income quartile		
Quartile 1	—	—
Quartile 2	1.00	0.98, 1.02
Quartile 3	0.99	0.97, 1.01
Quartile 4	0.99	0.96, 1.01
Primary payor^a^		
Medicare	1.32	**1.30, 1.33**
Medicaid	1.26	**1.24, 1.28**
Private insurance	0.69	**0.68, 0.70**
Self‐pay	0.83	**0.81, 0.85**
No charge	0.89	0.76, 1.04
Other	0.97	**0.94, 0.995**
Cancer type^a^		
Cancer of head and neck	1.20	**1.18, 1.22**
Cancer of esophagus	1.04	**1.01, 1.06**
Cancer of stomach	0.85	**0.83, 0.87**
Cancer of colon	0.90	**0.89, 0.91**
Cancer of rectum and anus	0.99	0.98, 1.01
Cancer of liver and intrahepatic bile duct	1.52	**1.49, 1.56**
Cancer of pancreas	0.85	**0.83, 0.86**
Cancer of other GI organs; peritoneum	0.86	**0.84, 0.88**
Cancer of bronchus; lung	1.09	**1.08, 1.1**
Cancer; other respiratory and intrathoracic	0.95	0.9, 1.01
Cancer of bone and connective tissue	0.74	**0.71, 0.76**
Melanomas of skin	1.17	**1.15, 1.19**
Other non‐epithelial cancer of skin	1.26	**1.23, 1.29**
Cancer of breast	1.00	0.99, 1.004
Cancer of uterus	0.99	0.97, 1.003
Cancer of cervix	1.08	**1.06, 1.11**
Cancer of ovary	0.88	**0.87, 0.89**
Cancer of other female genital organs	1.08	**1.05, 1.12**
Cancer of prostate	0.86	**0.85, 0.87**
Cancer of testis	1.16	**1.12, 1.2**
Cancer of other male genital organs	1.00	0.92, 1.09
Cancer of bladder	0.92	**0.91, 0.93**
Cancer of kidney and renal pelvis	1.00	0.99, 1.01
Cancer of other urinary organs	0.92	**0.87, 0.98**
Cancer of brain and nervous system	0.88	**0.86, 0.9**
Cancer of thyroid	1.00	0.98, 1.02
Hodgkin's disease	0.90	**0.87, 0.92**
Non‐Hodgkin's lymphoma	0.93	**0.92, 0.94**
Leukemias	0.89	**0.88, 0.9**
Multiple myeloma	0.82	**0.81, 0.84**
Cancer; other and unspecified primary	0.92	**0.89, 0.94**
Secondary malignancies	1.04	**1.02, 1.06**
Malignant neoplasm without specification of site	0.73	**0.72, 0.75**
Neoplasms of unspecified nature or uncertain behavior	1.16	**1.15, 1.18**
Maintenance chemotherapy; radiotherapy	0.54	**0.49, 0.58**

The bolded 95% CI values represent intervals which exclude the OR of 1 and therefore represent comparisons that are statistically significant.

Abbreviations: CI, Confidence interval; OR, Odds ratio.

^a^ Odds ratios for primary payor and cancer‐type categories compare each individual level to the combination of all other levels.

### 3.5 Demographic and clinical relationships with admission among primary MSUD

The relationship between selected variables with admission among cancer patients with a primary MSUD is shown in Table 4. Female patients (OR: 0.87, 95% CI: 0.85–0.90) and those with private insurance (OR: 0.90, 95% CI: 0.86, 0.93) or self‐pay (OR: 0.59, 95% CI: 0.55, 0.63) were less likely to be admitted to the hospital. In overall comparison to other cancer types, the three cancers most likely to be associated with admission are secondary malignancies (OR: 4.47, 95% CI: 4.12, 4.84), neoplasms of unspecified nature or uncertain behavior (OR: 2.39, 95% CI: 2.25, 2.53), and cancer of liver and intrahepatic bile duct (OR: 2.31, 95% CI: 2.14, 2.50).

**TABLE 4:** Adjusted odds ratio estimates for hospital admission in ED visits from cancer patients with primary MSUD during 2009–2018

Characteristics	OR	95% CI
Age at ED visit (per 10‐year increase)	0.99	0.98, 1.01
Sex		
Male	—	—
Female	0.87	**0.85, 0.90**
Income quartile		
Quartile 1	—	—
Quartile 2	0.96	0.91, 1.01
Quartile 3	0.99	0.93, 1.05
Quartile 4	0.99	0.91, 1.07
Primary payor^a^		
Medicare	1.30	**1.26, 1.34**
Medicaid	1.00	0.96, 1.04
Private insurance	0.90	**0.86, 0.93**
Self‐pay	0.59	**0.55, 0.63**
No charge	1.48	**1.13, 1.94**
Other	1.00	0.92, 1.09
Cancer type^a^		
Cancer of head and neck	1.03	0.97, 1.10
Cancer of esophagus	0.99	0.90, 1.10
Cancer of stomach	0.95	0.85, 1.06
Cancer of colon	0.84	**0.80, 0.88**
Cancer of rectum and anus	1.11	**1.01, 1.21**
Cancer of liver and intrahepatic bile duct	2.31	**2.14, 2.50**
Cancer of pancreas	0.86	**0.75, 0.99**
Cancer of other GI organs; peritoneum	0.70	**0.62, 0.79**
Cancer of bronchus; lung	0.82	**0.79, 0.86**
Cancer; other respiratory and intrathoracic	0.76	**0.58, 0.99**
Cancer of bone and connective tissue	0.83	**0.70, 0.99**
Melanomas of skin	1.10	**1.02, 1.18**
Other non‐epithelial cancer of skin	0.85	**0.78, 0.92**
Cancer of breast	0.87	**0.84, 0.90**
Cancer of uterus	1.12	**1.04, 1.20**
Cancer of cervix	0.98	0.93, 1.04
Cancer of ovary	0.90	**0.84, 0.97**
Cancer of other female genital organs	1.18	**1.02, 1.36**
Cancer of prostate	0.84	**0.80, 0.88**
Cancer of testis	0.91	**0.83, 0.997**
Cancer of other male genital organs	0.61	**0.42, 0.88**
Cancer of bladder	0.96	0.90, 1.02
Cancer of kidney and renal pelvis	1.13	**1.05, 1.21**
Cancer of other urinary organs	0.83	0.57, 1.22
Cancer of brain and nervous system	0.74	**0.67, 0.81**
Cancer of thyroid	0.96	0.90, 1.02
Hodgkin's disease	1.00	0.91, 1.10
Non‐Hodgkin's lymphoma	1.01	0.96, 1.07
Leukemias	0.98	0.92, 1.04
Multiple myeloma	1.29	**1.16, 1.43**
Cancer; other and unspecified primary	0.40	**0.37, 0.43**
Secondary malignancies	4.47	**4.12, 4.84**
Malignant neoplasm without specification of site	0.41	**0.35, 0.47**
Neoplasms of unspecified nature or uncertain behavior	2.39	**2.25, 2.53**
Maintenance chemotherapy; radiotherapy	0.16	**0.06, 0.39**

The bolded 95% CI values represent intervals which exclude the OR of 1 and therefore represent comparisons that are statistically significant.

Abbreviations: CI, Confidence interval; OR, Odds ratio.

^a^ Odds ratios for primary payor and cancer‐type categories compare each individual level to the combination of all other levels.

## 4 DISCUSSION

To the best of our knowledge, this study represents the largest to date to describe the frequency of mental disorders in cancer patients across multiple tumor subtypes in the emergency room setting. The findings from this study definitively demonstrate a high proportion of MSUD among cancer patient ED visits in the United States and identifies a subgroup of cancer types who may be particularly prone to mental illness. Although several retrospective, prospective, and systematic reviews have evaluated mental illness in the context of cancer,^6^, ^7^, ^8^, ^9^, ^10^, ^11^, ^12^ no previous studies have quantitatively analyzed the frequency of mental illness in cancer patients using such a large and robust dataset.

The finding in our study is that one in five visits among cancer patients who present to the ED have an associated MSUD, is consistent with what has been previously reported in the literature, albeit slightly lower in our study. Mehnert et al., evaluated mental disorders in 4020 cancer patients and demonstrated a 4‐week prevalence of any mental disorder of 32%.^13^ Singer et al., performed a meta‐analysis to assess the prevalence of mental health conditions in cancer patients and similarly found a prevalence of 32%.^7^ Similarly, Mitchell and colleagues performed a meta‐analysis to estimate the prevalence of mood disorders among cancer patients and found a prevalence of 29% for any type of mood disorder.^6^ One potential hypothesis for the lower proportion of MSUD in our study could be that these patients are less likely to seek urgent/emergency care.

Our study showed that both younger age and female sex were more likely associated with ED presentation for a MSUD. Previous studies have demonstrated that younger age is associated with high levels of psychological distress among adult cancer patients.^14^ Lang and colleagues evaluated psycho‐social distress among adolescent and young adult cancer survivors (AYA) and demonstrated higher rates of mood and anxiety disorders compared to older adult cancer survivors.^15^, ^16^ In addition, emotional distress has been shown to be higher among female compared to male cancer patients. Linden et al., screened 10,153 patients with cancer and demonstrated higher rates of anxiety and depression among female patients compared to male patients.^17^ Herschbach et al. evaluated psychosocial stress among 6365 cancer patients and found higher rates of psychological distress among female compared to male patients.^18^ In summary, our study describes the relationship of age and gender with psychosocial and mental distress and identifies a potential subgroup of patients for whom early screening for MSUD could be beneficial.

A unique finding in this study is the high rate of alcohol‐related disorders among cancer patients who presented to the ED for an MSUD. Not surprisingly, patients with tumors of the digestive tract had the highest percentage of alcohol‐related disorders. Previous studies have shown that 29% of adults in the United States have a history of an alcohol‐related disorder in their lifetime.^19^ Among cancer patients, alcohol use is associated with several adverse outcomes including prolonged postoperative morbidity, higher healthcare costs, and longer hospitalizations.^20^ In a recent study, Sanford et al., used the National Health interview Survey to examine alcohol drinking prevalence and showed that 56.5% of cancer patients were consumed alcohol and 21% participated in binge drinking.^21^ Collectively, these studies highlight the need for evidence‐based clinical interventions to reduce alcohol use in this population which may reduce healthcare costs and improve both disease and psychosocial outcomes.^22^, ^23^

Our study demonstrates high healthcare costs associated with mental health conditions among cancer patients, with a cost of over 3 billion dollars over a 10‐year period. A recent systematic review identified high total healthcare expenditure related to mental health disorders in cancer patients, with higher costs associated with cancer patients compared to non‐cancer patients.^24^ Very few studies have evaluated the cost of mental illness among cancer survivors, underscoring the need to urgently evaluate the economic and financial burden in this population.

Interestingly, we also found significant heterogeneity between different cancer subtypes and their predilection for MSUD. For example, primary diagnosis of alcohol‐related disorders were more likely among patients with cancer of the aero‐digestive tract, whereas anxiety and depressive disorders were more likely among patients with cancers of the thyroid, lymphoid tissue, breast, uterus, ovary, and cervix. These findings are important as they may allow oncologists and their colleagues to direct and tailor psychosocial interventions and resources to the specific subtype of cancer.

Our study has several limitations that must be acknowledged. First, specific data on tumor stage and biology are not present in the NEDS. Therefore, it is unclear if advanced stage or metastatic disease may be associated with the development of an MSUD. Other studies suggest greater symptom burden and higher costs among this group of patients.^25^, ^26^ Our database only identifies patients who presented to the ED, so the true prevalence of mental illness among cancer patients is likely higher. Next, the data in NEDS are reported as an ED visit rather than individual patient data, which could bias the data toward patients who have more frequent admissions. Finally, data regarding the specific therapy for each patient's cancer diagnosis is not provided in the database, which prevents our ability to determine if any specific therapy may influence the rate of MSUD.

In summary, our study demonstrates a high proportion of mental disorders among visits to the ED from cancer patients, with heterogeneity in the proportion of specific types of mental disorders by tumor subtype. Our findings underscore the importance of addressing mental disorders early in the course of cancer diagnosis which may provide an opportunity for interventions to improve psychosocial and cancer‐related outcomes, reduce ED visits, and reduce financial burden in this population.

## AUTHOR CONTRIBUTIONS

**Sujith Baliga:** Conceptualization (lead); data curation (lead); formal analysis (lead); investigation (lead); methodology (lead); project administration (lead); resources (equal); software (lead); writing – original draft (lead); writing – review and editing (lead). **Brett Klamer:** Data curation (equal); formal analysis (equal); methodology (equal); resources (lead); software (lead); visualization (lead). **Joshua David Palmer:** Writing – review and editing (supporting). **Sharla Wells‐Di Gregorio:** Writing – review and editing (supporting). **Sachin Kale:** Writing – review and editing (supporting). **Marcelo Bonomi:** Writing – review and editing (supporting). **Matthew Old:** Writing – review and editing (supporting). **James W Rocco:** Writing – review and editing (supporting). **Dukagjin Blakaj:** Investigation (supporting); methodology (supporting); visualization (supporting); writing – review and editing (equal).

## FUNDING INFORMATION

None

## CONFLICT OF INTEREST

None.

## Data Availability

The data is public and available from the NEDS website for a fee.
